# A Break in the Continuum: Analyzing the Gap in Particle Exposure Research

**DOI:** 10.1289/ehp.118-a543b

**Published:** 2010-12

**Authors:** Tanya Tillett

**Affiliations:** **Tanya Tillett**, MA, of Durham, NC, is a staff writer/editor for *EHP*. She has been on the *EHP* staff since 2000 and has represented the journal at national and international conferences

Researchers have examined the effects of fine particulate matter (PM_2.5_) doses spanning nearly three orders of magnitude. Cardiovascular disease risks have been documented for active smoking exposures at the higher end of that continuum and for environmental tobacco smoke (ETS; sometimes called secondhand smoke) and ambient air pollution exposures at the lower end. However, there is a distinct lack of information on the cardiovascular risks of exposures in the middle range, which are experienced by hundreds of millions of people who burn biomass and coal indoors. A new commentary analyzes the implications of this crucial knowledge gap **[*EHP*** **118(12):1643–1645; Smith and Peel]**.

The authors note that plots of average inhaled doses of PM_2.5_ and associated cardiovascular health risks in the literature reveal a highly nonlinear exposure response. That is, exposure to ETS and ambient air pollution yield higher risks of heart disease than might be expected on the basis of exposure risks of active smokers.

Analysis of three intermediate estimated PM_2.5_ doses (inhaling 100 mg/day for a light smoker, 10 mg/day for a resident exposed to smoke from biomass burning, and 1 mg/day for someone exposed to ETS) similarly indicated that small PM_2.5_ dose reductions in populations with relatively low levels of exposure may achieve greater health gains than more drastic reductions in populations with high levels of exposure. Comparing a theoretical population of smokers with the same number of nonsmokers living in a heavily polluted city, this suggests similar numbers of cardiovascular deaths—or possibly more—may be prevented in the nonsmokers by adopting strict ambient PM_2.5_ reduction measures than by convincing the smokers to quit smoking. This counterintuitive idea could have major policy ramifications, so the authors urge further examination of these relationships.

The gap in studies of exposures greater than those from ETS but less than those from active smoking reflects a dose range of 1–20 mg/day. Filling in the missing information for this gap should receive research priority, the authors write, because of the potentially large cardiovascular disease health burden it represents, particularly for the people worldwide who burn biomass for household cooking and heating.

## Figures and Tables

**Figure:**
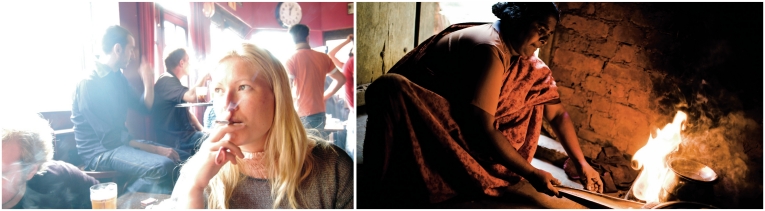
The health effects of particulate exposure through active and passive smoking have been studied extensively. Less is known about similar exposures that result from indoor biomass and coal burning, a part of daily life for hundreds of millions of people worldwide.

